# Micro-Structure Determines the Intrinsic Property Difference of Bio-Based Nitrogen-Doped Porous Carbon—A Case Study

**DOI:** 10.3390/nano10091765

**Published:** 2020-09-07

**Authors:** Yingfang Jiang, Yanxia Liu, Yagang Zhang, Yidan Chen, Xingjie Zan

**Affiliations:** 1 School of Materials and Energy, University of Electronic Science and Technology of China, Chengdu 611731, China; jiangyingfang@ms.xjb.ac.cn (Y.J.); liuyanxia@ms.xjb.ac.cn (Y.L.); 2 Department of Chemical and Environmental Engineering, Xinjiang Institute of Engineering, Urumqi 830026, China; chenyd2020@ms.xjb.ac.cn; 3 Xinjiang Technical Institute of Physics and Chemistry, Chinese Academy of Sciences, Urumqi 830011, China; zanxj@ms.xjb.ac.cn

**Keywords:** cottonseed hull, cattail, nitrogen doped porous carbon, structure-property relationship, performance

## Abstract

Biomass-derived porous carbon materials have drawn considerable attention due to their natural abundance and low cost. In this work, nitrogen-doped porous carbons with high nitrogen content and large surface areas were designed and prepared from cottonseed hull and cattail. The two plant-based biomass compositions are similar, but the structures are very different, generating distinctly different property and performance of the prepared carbon materials. NRPC-112 has good electrochemical properties, while CN800 has good adsorption properties. By comparing the microstructure differences between the two starting materials, it was found that the structure of the raw materials would significantly affect the properties and performance of the materials. The work provided an important theoretical basis and reference for the selection of bio-resources for preparing carbon material. It is also important for choosing the appropriate synthesis method, process optimization, and application scenarios.

## 1. Introduction

Biomass porous carbon materials have attracted considerable attention for their potential applications in chemical catalyst, energy and fuels, water treatment and retention, and specialty functional materials [1,2]. This is ascribed to their superior properties such as renewable features, low cost, low environmental impact, and sustainable availability [3]. It is believed that the biomass is one of the most feasible ways to prepare carbon materials [4].

Efforts have been devoted to preparing porous carbon from different carbon sources. Various carbon precursors [5,6,7,8,9,10,11,12,13,14] including plants (cotton, rice husk, soya), animals (fish gill, pig bone), microorganism (yeast cells) have been used for preparing porous carbon, and have a wide application in many fields, such as active materials for energy storage, absorbent agents, and biomedical use.

Biomass based carbon materials is an important area in green chemistry. Various materials and strategies have been developed for the synthesis of biomass based materials [15,16,17]. Nevertheless, the complexity of the raw materials and the diversity of the preparation methods lead to significant differences in the property and performance of the carbon materials. With different biomass precursors, the performances of the prepared porous carbon materials are quite different. What is the main reason behind these property and performance differences? Extremely few relevant literatures have addressed this key issue so far.

Along this line, in order to address the key fundamental question, we specifically choose two bio-resource based precursors: cottonseed hull and cattail, respectively as starting material for preparation of nitrogen doped porous carbon. Their main components are lignin, cellulose and hemicellulose. Cottonseed hull is the outer shell of cottonseed, with a relatively hard and strong texture while cattail has a high lignin and cellulose content [18,19,20,21], which is structurally very loose and fluffy with naturally developed abundant pore structures. The purpose of this work was to synthesize porous carbons with cottonseed hull and cattail as raw materials. By systematically comparing the chemical composition, microscopic structure of two precursors, as well as further characterizing the adsorption property and electrochemical performance of the prepared carbon materials, the influence of the starting materials’ structural differences on the property of bio-based nitrogen-doped porous carbon was explored and discussed.

## 2. Materials and Methods 

### 2.1. Materials

Cottonseed hull was smashed by grinder into particles with 200 meshes sieve before use. Potassium hydroxide (Zhiyuan Company, Tianjin, China), urea (Kermel Company, Tianjin, China), hydrochloric acid (Zhiyuan Company, Tianjin, China) were all of analytical grade. Cattail was washed repeatedly with ethanol, and then dried in a vacuum drying box at 60 °C for later use. Potassium carbonate (K_2_CO_3_), urea and ethanol were all analytically pure and all from Kermel Company, cottonseed hull and cattail all came from Xinjiang.

### 2.2. Synthesis of Nitrogen-Enriched Porous Carbons

Cottonseed hull: a mixture of cottonseed hull (4.0 g), KOH (4.0 g) and urea (8.0 g) (the mass ratio was 1:1:2) were mixed with 30 mL deionized water in a 100 mL flask and stirred at ambient temperature (15 °C) for 2 h. Then the mixture was transferred into a porcelain boat and heated to 800 °C for 2 h with a heating rate of 5 °C min^−1^ under a nitrogen atmosphere. After cooling down to ambient temperature, the obtained sample was immersed into the HCl solution (2.0 mol L^−1^) and stirred for 1 h, and then washed repeatedly with ultra-pure water until the pH value reached 7. Finally, the black sample was dried under vacuum at 60 °C for 12 h and denoted as NRPC-112, for comparison, cottonseed hull: KOH: urea was 1:0:0 was labelled as CH-100.

Cattail: First, 6 g potassium carbonate and 6 g urea were added into 50 mL 20% ethanol solution, then 6 g of dried cattail was added in solution, stirred at room temperature for 2 h, then the soaked cattail was placed in a ceramic boat in a tubular furnace, under the protection of nitrogen atmosphere, heated at a heating rate of 5 °C min^−1^, from room temperature to 800 °C min^−1^. After holding for 2 h, the sample was cooled to room temperature at the same rate. Then the sample was washed to neutral. It was dried in a vacuum drying box at 60 °C for 12 h, the sample was named CN800, for comparison, cattail: K_2_CO_3_: urea was 1:0:0 was labelled as C800.

### 2.3. Materials Characterization

X-ray diffraction (XRD) patterns of NRPC-112 and CN800 were obtained on an XRD analyzer (D8-Advance, Bruker AXS, Karlsruhe, Germany) equipped with a diffracted-beam monochromator using Cu Kα radiation (50 kV, 40 mA). The Raman spectra were obtained using a Raman spectroscopy (Horiba Scientific, Paris, France) with a 532 nm blue laser beam. The microstructures of all samples were observed with field emission scanning electron microscopy (FE-SEM, ZEISS, Jena, Germany) and transmission electron microscopy (H-600, Hitachi, Japan). Surface area was tested by Brunauer-Emmer-Teller (BET) method with use of the nitrogen absorption/desorption measurement (V-Sorb 2800P, Beijing, China). All samples were degassed in vacuum at 200 °C for 5 h prior to sorption experiments. X-ray photoelectron spectroscopy (XPS) was carried out on an XPS (ESCALAB 250Xi, Thermo, MA, USA), with a monochromatic Al Kα as an excitation source.

### 2.4. Electrochemical Measurements

Electrochemical tests were carried out on a CHI660E electrochemical workstation (Chenhua, Shanghai, China) at ambient temperature. The working electrodes were made according to the following process: 90 wt% active material, 5 wt% black carbon, and 5 wt% polytetrafluoroethylene (PTFE) were mixed with ethanol. Then, the mixture was coated on a titanium mesh (1 cm × 1 cm), followed by pressing at a pressure of 15 MPa for one minute and finally dried at 60 °C in vacuum oven for 2 h. The loading mass of the active material on each electrode was 5.0 mg.

In a three-electrode system, a platinum slice was used as the counter electrode and Hg/HgO was used as the reference electrode in 6 M KOH. Galvanostatic charge/discharge (GCD) were carried out in the potential range of −1 V to 0 V at scan rates of 5–100 mV s^−1^.

(1) $$C=\frac{I\times \Delta t}{m\times \Delta V}$$

### 2.5. Pollutants Removal

In order to test the absorption capacity of NRPC-112 and CN800, the adsorption studies for triclosan (TCS) were carried out in acetonitrile, NRPC-112 (7 samples, 5 mg each) and CN800 (7 samples, 5 mg each) were added to 5 mL of TCS solution with an initial concentration from 100 to 2000 mg L^−1^, the mixtures were shaken continually for 2 h. Each sample was passed through a Teflon filter to separate particles from the supernatant. Residual concentrations of TCS in filtrate were quantified by measuring the UV absorbance at 280 nm. The adsorption capacity can be calculated according to the following Equation (2).

(2) $$Q_{t}=\frac{\left(C_{0}-C_{t}\right)\;\times \;V}{m}$$

where *Q_t_* (mg g^−1^) is adsorption capacity of NRPC-112 at different time intervals; *C*_0_ (mg L^−1^) and *C_t_* (mg L^−1^) are the initial and residual concentrations of TCS, respectively; *V* (L) is the volume of TCS solution; and *m* (g) is the mass of the absorbent.

The test conditions for the other two substances are as mentioned above. The test conditions for different substances are different, these test conditions are:

Dichlorophene (DCP) test conditions: ethanol solvent, absorption wavelength 286 nm; roxarsone (ROX) test conditions: methanol solvent, absorption wavelength 325 nm.

## 3. Results and Discussion

### 3.1. Selection of Raw Materials

**Figure 1:**
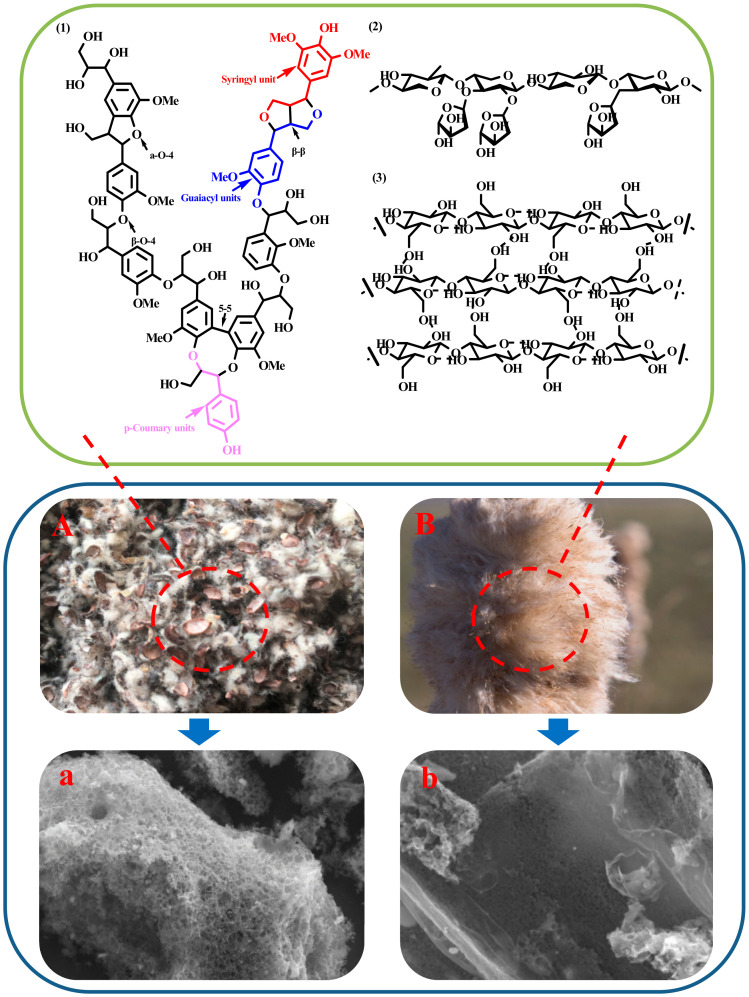
Carbon materials from natural bio-resource (**A**) cottonseed hull; (**B**) cattail; (**a**) porous carbon from A; (**b**) porous carbon from B; (**1**) Lignin; (**2**) hemicelluloses; (**3**) cellulose.

Cattail and cottonseed hull are biomass materials, the main components of which are cellulose, lignin and hemicellulose [22,23,24]. As shown in Figure 1, key questions are: What is the difference in performance between biomass with the same or similar composition but different structures after carbonization and activation at the same temperature? Can rational yields and performance be obtained under suitable carbonization and activation conditions? What is the difference in the structure and properties of the carbon materials produced? The microstructures of carbon materials prepared by different raw materials vary greatly, as shown in Figure 1. The carbon material prepared from cottonseed hulls containing continuous, interconnected, porous structure, while the carbon material prepared from cattails has a tight pore structure. Different microstructures also affect the properties of materials. Therefore, it is very important to select suitable raw materials.

### 3.2. Selection of Preparation Methods

**Figure 2:**
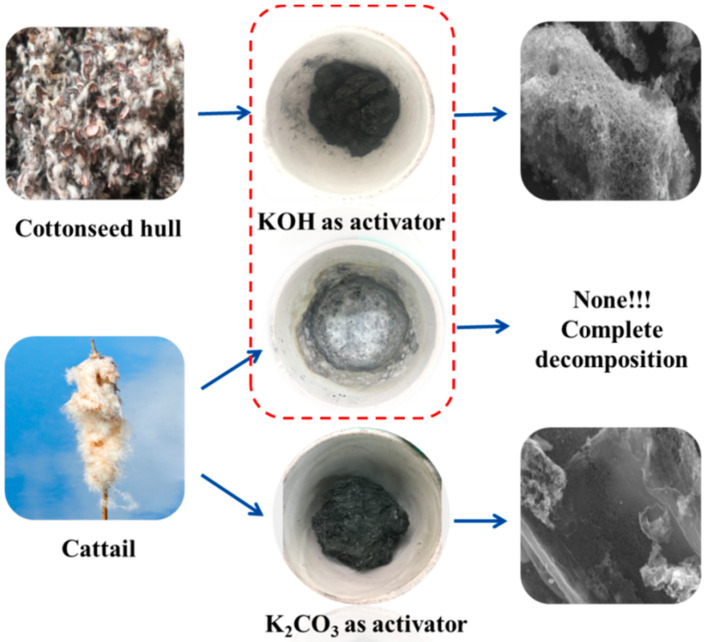
Preparation of porous carbon materials with different activator.

When biomass is used as raw materials to prepare nitrogen-doped porous carbon materials, the choice of activator is very important [23,25,26]. The material prepared from the cottonseed hull with potassium hydroxide as the activator has continuous, interconnected porous structure, while with the same method for cattail, when strong alkali KOH was used as activator for impregnation, strong alkali etching destroyed the structure of the cattail. After carbonization at high temperature, cattail was completely decomposed, and porous carbon materials cannot be obtained, as shown in Figure 2. Therefore, cattail materials containing a large amount of fibrous structure will be dissolved in strong alkali. Strong alkali is not ideal activator for raw materials cattail. When weak alkali potassium carbonate is chosen as activator, nitrogen-doped porous carbon materials can be obtained from cattail after impregnation and carbonization. As shown in Figure 2, the etching effect of weak alkali is poor, which will lead to smaller specific surface area of carbon materials prepared by cattail. Therefore, different methods should be selected for different materials.

### 3.3. Characterization of Material Properties

The yield, elemental analysis and XPS surface elemental analysis of cattail and cottonseed hulls after carbonization and activation at 800 °C are listed in Table 1 below. It can be seen from the table that at the same carbonization temperature, the carbon yield of NRPC-112 prepared from cottonseed hull is 15.4%, and the yield from cattail is 11.7%. The results show that the yields of materials after carbonization at the same temperature were significantly different, so it is very important to select the appropriate raw materials.

As seen from Table 1, it was observed that CH-100 exhibited a nitrogen content of 2.15%, and NRPC-112 consisted of carbon (64.43%), nitrogen (8.98%), hydrogen (1.40%); meanwhile C800 exhibited a nitrogen content of 1.24%, and CN800 consisted of carbon (65.98%), nitrogen (7.52%), hydrogen (3.17%); which implied urea was favorable for generating N rich carbon materials. The content of nitrogen on the surface of samples was in agreement with that of elemental analysis, which further indicated that the nitrogen was successfully doped into the carbon matrix.

**Figure 3:**
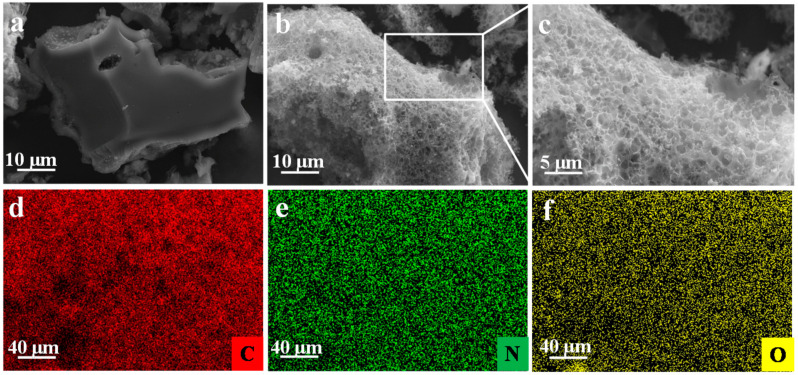
SEM images of CH-100 (**a**), NRPC-112 (**b**,**c**), and the corresponding EDX mapping of (**d**) C, (**e**) N, (**f**) O.

The morphology and structure of samples were observed by scanning electron microscopy (SEM). The morphology of CH-100 and NRPC-112 were depicted in Figure 3. The SEM images as shown in Figure 3a, CH-100 exhibited smooth surface and dense structure with very limited pores and channels. It also can be seen from SEM images (Figure 3b,c) that NRPC-112 containing interconnected, continuous, porosis porous structure. The crosslinked porous structure could provide the channel of electrode ions diffusion and transmission [27]. Furthermore, in order to investigate the elemental distribution of C, O, and N, energy-dispersive X-ray spectroscopy (EDS) mapping was performed and the results were shown in Figure 3d–f. All elements displayed a homogeneous distribution on the surface of the sample, indicating that N was successfully incorporated into NRPC-112 matrix.

**Figure 4:**
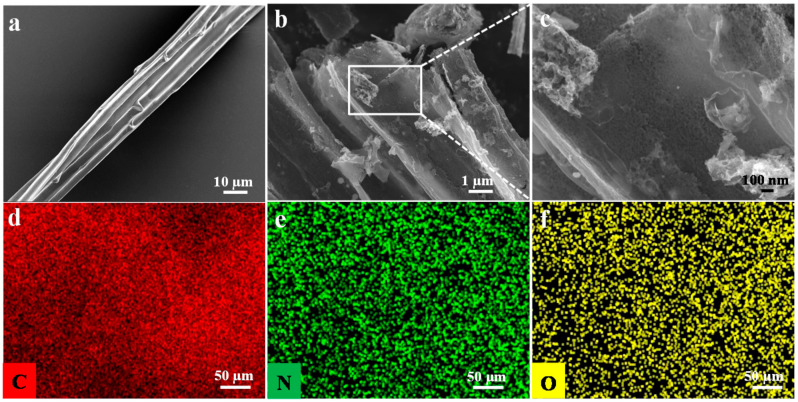
SEM images of C800 (**a**), CN800 (**b**,**c**), and the corresponding EDX mapping of (**d**) C, (**e**) N, (**f**) O.

Along this line, the morphology of C800 and CN800 observed in Figure 4. As shown in Figure 4a, the SEM image of C800 showed smooth, tubular, hollow structure. Compared with C800, the obtained CN800 as shown in Figure 4b,c appeared hole on the tube wall, which still maintains the original hollow structure. As shown in Figure 4d–f, C, N, and O elements displayed a homogeneous distribution on the surface of the sample, indicating that N was successfully incorporated into CN800 matrix. The above results show that the carbon material after carbonization and activation at high temperature still maintain the original structure, and the strong base is conducive to the formation of materials containing loose porous structures.

**Figure 5:**
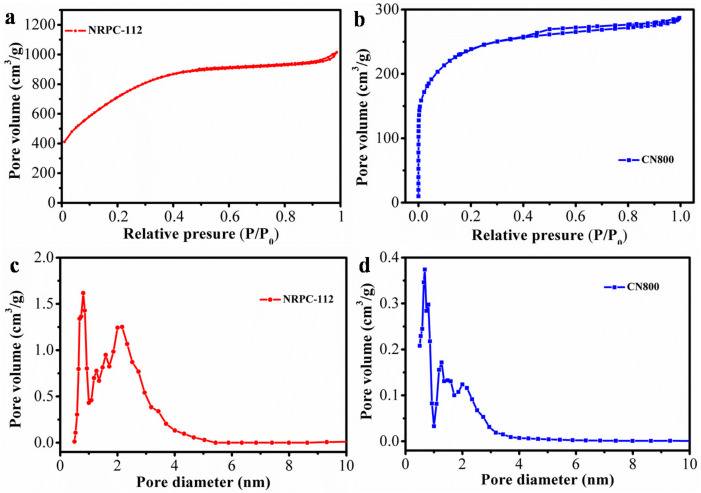
(**a**) N_2_ adsorption-desorption isotherm of NRPC-112, (**b**) N_2_ adsorption-desorption isotherm of CN800, (**c**) pore width distribution of NRPC-112 obtained by DFT method, (**d**) pore width distribution of CN800 obtained by DFT method.

N_2_ adsorption/desorption curves were used to characterize the pore structure of the prepared carbon materials. The adsorption-desorption isotherm and pore size distribution were shown in Figure 5. Table 2 summarized the specific surface area and pore size distribution of prepared carbon materials NRPC-112 and CN800. As shown in Figure 5a,b, according to the classification of IUPAC, NRPC-112 and CN800 show typical type I isotherms, which means that the prepared carbon materials mainly have microporous structure. Figure 5c–d shows that the pore sizes of NRPC-112 and CN800 were mainly in the range of 0.1 to 2 nm, which indicated that the two materials mainly possess micropores. These results indicated that both KOH and K_2_CO_3_ can effectively improve the porosity and increase the specific surface area of carbon materials. Compared with K_2_CO_3_, the strong base KOH is more helpful for the preparation of porous carbon materials.

It was found in Table 2 that the cottonseed hulls (without urea and KOH) carbonized at 800 °C only had specific surface area of 15 m^2^ g^−1^ and larger average pore diameter, while the NRPC-112 had a much larger specific surface area (2573 m^2^ g^−1^) and smaller average pore diameter (1.99 nm). Comparing the above results, the specific surface area of CN800 was 865 m^2^ g^−1^ and average pore diameter was 2.05 nm, the surface area of the raw material C800 was 21 m^2^ g^−1^, indicating that both of these raw materials have been successfully converted to porous carbon materials. Besides, the specific surface area of NRPC-112 is larger than CN800. This implied that strong base etching is beneficial for forming microporous structure; it is advantageous to obtain abundant pore structure with larger specific surface area.

**Figure 6:**
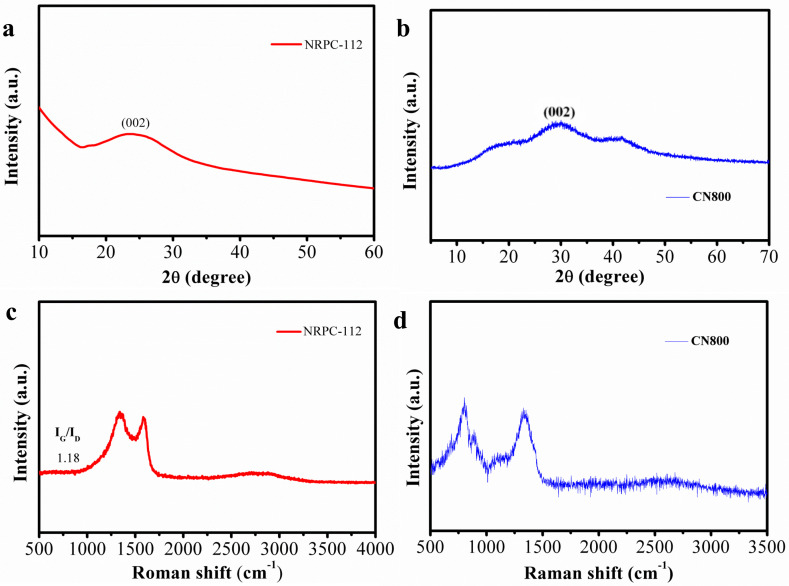
(**a**) XRD patterns of NRPC-112, (**b**) Raman spectra of NRPC-112, (**c**) XRD patterns of CN800, (**d**) Raman spectra of CN800.

The crystal structure and graphitization degree of NRPC-112 and CN800 were analyzed by X-ray diffraction and Raman spectroscopy (as shown in Figure 6). As shown in Figure 6a,b, the diffraction peaks appear at 2θ = 20–30°, which were assigned to the (002) plane of graphitic carbon. The position (angle) of the (002) band was believed to be related to the inter-lamellar spacing [27], indicating that NRPC-112 and CN800 have amorphous structure and the degree of graphitization is low. The Raman spectra show that the I_G_/I_D_ value of NRPC-112 is 1.18 as shown in Figure 6c. Raman spectra of CN800 (Figure 6d) show that the two characteristic peaks occur in 1350 cm^−1^ (G-band) and 785 cm^−1^ (D-band). While for NRPC-112, those two peaks occur in 1580 cm^−1^ (G-band) and 1300 cm^−1^ (D-band). The D-band is from the vibration of carbon within defects. While the G-band is from the vibration of in plane sp^2^ hybridized carbon in microcrystalline structure. The D-band is related to the degree of crystal defects, while the G-band represents the microcrystalline structure. The ratio of G-peak to D-peak (I_G_/I_D_) reflects the degree of defect and graphitization. The I_G_/I_D_ value of CN800 is 1.08, which indicates that CN800 doped with N and O has typical amorphous structure and relatively low graphitization degree. It further shows that the prepared carbon materials have amorphous structure, there are defects in the material, and the existence of defective structure is conducive to ions transmission and the adsorption of pollutants.

**Figure 7:**
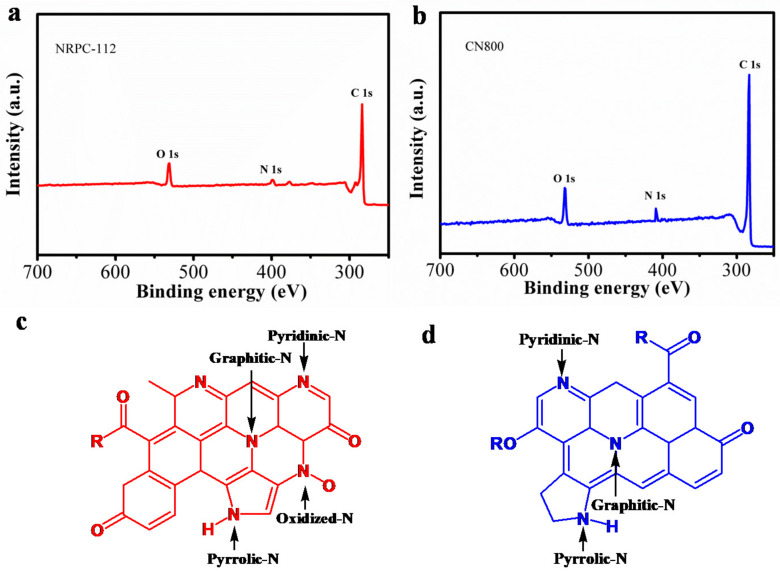
(**a**) XPS survey spectra of NRPC-112, (**b**) XPS survey spectra of CN800, (**c**) different of N and O in NRPC-112, (**d**) different of N and O in CN800.

**Figure 8:**
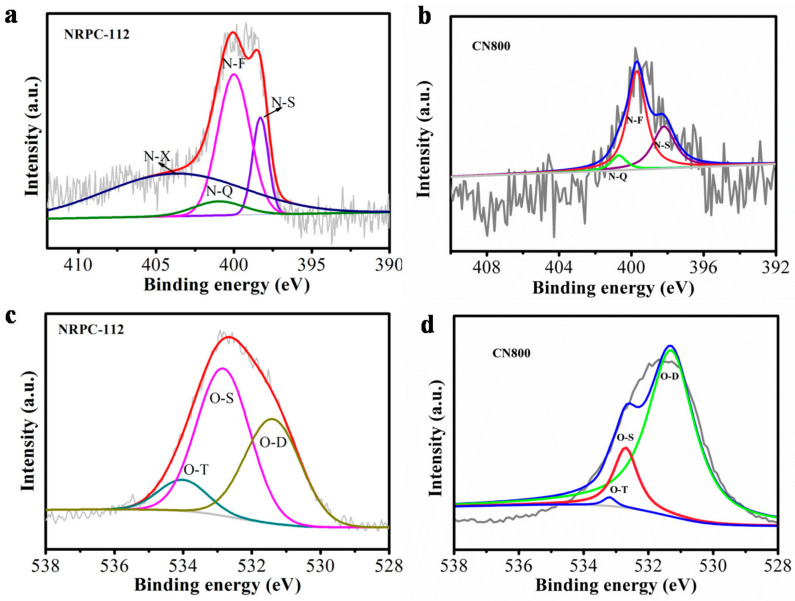
XPS spectra of the as-prepared carbon samples (**a**) N 1s of NRPC-112, (**b**) N 1s of CN800, (**c**) O 1s of NRPC-112, (**d**) O 1s of CN800.

The surface chemical properties of NRPC-112 and CN800 were characterized by XPS. As shown in Figure 7a,b, three characteristic peaks were observed at 285 eV, 400 eV and 533 eV, which correspond to C 1s, N 1s and O 1s, respectively [28,29,30]. Then the types of nitrogen and oxygen were distinguished with high resolution scans (Figure 8).

The high resolution scan of N 1s of NRPC-112 as shown in Figure 8a, there were four types of N signals, they were pyridine-N (N–S), pyrrole-N (N–F), graphite-N (N–Q) and oxidized-N (N–X), corresponding to peaks at 398.2 eV, 399.7 eV, 400.7 eV and 402.8 eV, respectively [31]. The high-resolution scans of N 1s of CN800 as shown in Figure 8b, there were three types of N signals, they were pyridine-N (N–S), pyrrole-N (N–F), graphite-N (N–Q). The oxidized nitrogen N (N–X) was not observed, probably due to the different choice of activator. Potassium hydroxide has a strong basicity, which can generate more pore structure during the activation process, and cause more defects, which is beneficial to nitrogen atoms embedded into carbon matrix; while potassium carbonate has weak alkalinity and weak activation. The prepared carbon material has much less pore structure and small specific surface area, so the ability to combine with nitrogen element is weak.

The high-resolution XPS scan of O1s of NRPC-112 as shown in Figure 8c, three types of O signals, namely C=O oxygen or quinine-type groups (O–D), C–OH phenol groups/C–O–C ether groups (O–S) and O=C–O (carboxylic group, chemisorbed oxygen)/or water(O–T) were observed, which were ascribed to the peaks at 531.3, 532.7 and 534.2 eV, respectively [31]. Similarly, the high-resolution XPS scan of the O1s of CN800 as shown in Figure 8d, three types of O signals were observed. It was believed that the N could facilitate ion transfer to the carbon layers, while enhancing the conductivity of the materials [32]. Furthermore, different types of O within the carbon materials could enhance the surface wettability, and all materials had large amount of O–D, which implied that they had low resistance to ion transfer between the electrolyte [33]. O–S was electrochemically active, which was beneficial for promoting pseudocapacitance of the electrode. O–T also facilitated good performances in the electrochemical tests [34]. 

N and O in different chemical states within NRPC-112 are schematically shown in Figure 7c, N and O in different chemical states within CN800 is shown in Figure 7d. Their possible structures indicated the successful incorporation of nitrogen atoms. What is the difference in performance between the two materials with different structures? The electrochemical and adsorption properties of the two materials were tested and compared.

### 3.4. Electrochemical Properties

After the successful synthesis of the nitrogen-doped porous carbons with appropriate pore structures, high specific surface areas and nitrogen content, the electrochemical performance of the prepared NRPC-112 and CN800 were assessed. The electrochemical properties of NRPC-112 and CN800 were evaluated in a three-electrode system using 6 M KOH solution as electrolyte.

**Figure 9:**
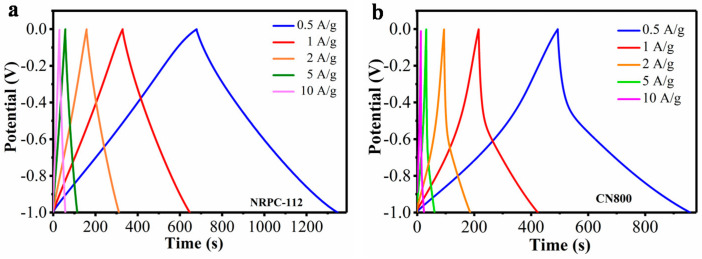
(**a**) Galvanostatic charge/discharge (GCD) curves at 0.5–10 A g^−1^ of NRPC-112, (**b**) GCD curves at 0.5–10 A g^−1^ of CN800.

Galvanostatic charge/discharge (GCD) profiles of NRPC-112 and CN800 were shown in Figure 9. NRPC-112 electrode was chosen to evaluate its supercapacitors properties due to its superior electro-chemical performance. Figure 9a showed the GCD curves of NRPC-112 at various current densities. It was found that the GCD curves of NRPC-112 showed quasi-isosceles shape as the current density increasing from 0.5 A g^−1^ to 10 A g^−1^, illustrating that the sample had good coulombic efficiency and decent double-layer capacitive performance. Furthermore, from the GCD results at current density of 0.5 A g^−1^, the NRPC-112 exhibited the largest gravimetric capacitance *(C_g_)* values (340 F g^−1^). Figure 9b showed the GCD curves of CN800 at various current densities. It was found that the GCD curves of CN800 showed irregular shape as the current density increasing from 0.5 A g^−1^ to 10 A g^−1^, illustrating that the charge/discharging process is not a completely equivalent process. The resistance of ion movement is different during the charge and discharge process. In addition, from the GCD results at current density of 0.5 A g^−1^, the CN800 had the largest gravimetric capacitance (*C_g_*) values (246 F g^−1^).

Table 3 showed the specific capacitance at various current densities. It showed that the specific capacitance of NRPC-112 was 340, 329, 316, 287, 273 F g^−1^ at the current density of 0.5, 1, 2, 5, 10 A g^−1^, respectively, implying the rate capability of ~80% retention (the ratio of the specific capacitance at the current density of 10 A g^−1^ and 0.5 A g^−1^); while the specific capacitance of CN800 was 246, 216, 189, 158, 111 F g^−1^ at the current density of 0.5, 1, 2, 5, 10 A g^−1^, respectively, indicating the rate capability of ~45% retention. The main reasons for these differences are as follows: firstly, the structure of the cottonseed hull is dense and has no pores, and the NRPC-112 has continuous interconnected and porous structure, micropores could provide abundant active sites for ion storage, while micropores could promote fast ion transmission [35]. Moreover, the pores interconnected to each other. This forms a channel for ion diffusion, which is beneficial to the improvement of electrochemical performance. Secondly, the cattail itself has a hollow tubular structure. The prepared porous carbon material only has holes in the tube wall, and there is no unblocked channel for ion diffusion, so the charge and discharge performance is relatively poor. In conclusion, the structure of the biomass itself has a great influence on the properties of the prepared porous carbon material.

### 3.5. Adsorption Performance Test

Chlorophenols are aromatic compounds. They are important chemical raw material, intermediate and organic solvent and most of the chlorophenols are highly toxic, difficult to biodegrade, and have “three-way” (carcinogenic, teratogenic, mutagenic) effects [36,37]. Due to the long degradation cycle, they are difficult to remove in a natural environment.

Dichlorophene (DCP) is often used as a biocide in industrial production. It can kill bacteria in water. It is widely used in oil refining, the chemical industry and other industrial production. It can cause damage to the original organisms in the water [38]. Triclosan (TCS) is a highly effective spectral fungicide, widely used in toothpaste, hand sanitizer, soap and deodorant, but it has many side effects. One is that it can be converted into dioxins under the condition of light, which can turn into toxic substances. The other is that the excessive intake of TCS can lead to malformation and liver damage [2]. Roxarsone (ROX) is a feed additive for livestock and poultry. It can promote animal growth and prevent malaria. However, Roxarsone is an arsenic-containing additive, and a carcinogenic substance. Therefore, it is of paramount importance to develop functional materials for the recognition and removal of emerging pollutants [39].

**Figure 10:**
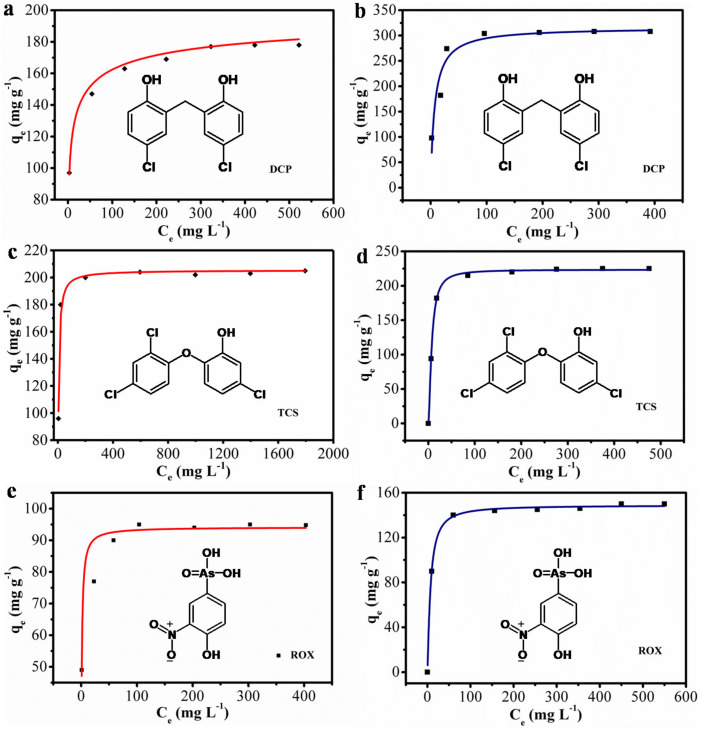
Equilibrium adsorption isotherms of NRPC-112 (**a**) dichlorophene (DCP), (**c**) triclosan (TCS), (**e**) roxarsone (ROX), Equilibrium adsorption isotherms of CN800 (**b**) DCP, (**d**) TCS, (**f**) ROX.

The adsorption results for the three pollutants are shown in Figure 10. NRPC-112 and CN800 materials have adsorption on all three substances, but the adsorption capacity was different. The adsorption isotherm of NRPC-112 on DCP is shown in Figure 10a, and the adsorption isotherm of CN800 on DCP is shown in the Figure 10b. With increasing concentration of DCP, the DCP adsorption capacity at the time of adsorption equilibrium of NRPC-112 and CN800 both gradually increased, while CN800 exhibited the higher capacity on DCP; the adsorption isotherm of NRPC-112 on TCS is shown in Figure 10c, the adsorption isotherm of CN800 on TCS is shown in Figure 10d, the adsorption capacity of the NRPC-112 and CN800 for TCS is not very different. In addition, the adsorption isotherm of NRPC-112 on ROX is shown in Figure 10e, the adsorption isotherm of CN800 on ROX is shown in Figure 10f, and the adsorption capacity of CN800 for ROX is higher than NRPC-112. From the adsorption results of the three substances, it can be seen that the CN800 exhibits higher adsorption performance. What are the reasons for this adsorption performance?

The specific adsorption capacity of NRPC-112 and CN800 is shown in Table 4, the adsorption capacity of DCP on NRPC-112 is 174 mg g^−1^, and that of CN800 is 308 mg g^−1^. The adsorption capacity of TCS on NRPC-112 is 205 mg g^−1^, and that of CN800 prepared from cattail is 225 mg g^−1^; and the adsorption capacity of ROX on NRPC-112 is 92 mg g^−1^, and CN800 is 150 mg g^−1^. Based on the above results, the adsorption capacity of CN800 prepared from the cattail was better than that of NRPC-112 prepared from the cottonseed hull; the results can be explained as follows. Firstly, the special structure of cattail facilitates the adsorption of pollutants, the morphology of CN800 maintains the original hollow tubular structure, and holes are in the wall of the pipe, so the pollutants could efficiently enter the cavity through holes, which is beneficial for the capture of the contaminant, so its adsorption performance is better. Secondly, NRPC-112 and CN800 materials have better adsorption performance for chlorine-containing contaminants. NRPC-112 and CN800 provided various amine groups which could form hydrogen bonds with an O atom and interact with hydroxyl groups through acid-base interaction. In summary, the results implied that the prepared carbons hold great application potential for the recognition and removal of chlorine-containing contaminants.

## 4. Conclusions

Two bio-resource based precursors: cottonseed hull and cattail were chosen as starting materials. Nitrogen-doped porous carbons with high nitrogen content and large surface areas were designed and prepared from cottonseed hull and cattail. By systematically comparing the chemical composition and microscopic structure of two precursors, as well as further characterizing the adsorption property and electrochemical performance of the prepared carbon materials, the influence of the starting materials’ structural difference on the property of bio-based nitrogen doped porous carbon was explored and discussed. The chemical compositions of the two bio-based precursors are similar but the structures are very different, generating distinctly different properties and performance of the prepared carbon materials. The results showed that NRPC-112 has good electrochemical properties, while CN800 has good adsorption properties. The choice of raw materials and preparation methods, and the structure of the raw materials would affect the properties of the materials. The work provided an important reference guide for the preparation of carbon materials, especially for the choice of methods, process optimization and application development.

## Figures and Tables

**Table 1:** Yields, elemental analysis, X-ray photoelectron spectroscopy (XPS) analysis of the C800 and CN800.

Material	Yield (%)	Elemental Analysis			XPS		
		C (%)	N (%)	H (%)	C (%)	N (%)	O (%)
CH-100	N/A	76.44	2.15	1.23	78.45	1.84	19.72
NRPC-112	15.4	64.43	8.98	1.40	80.45	8.44	10.81
C800	N/A	78.82	1.24	2.56	84.61	1.16	14.23
CN800	11.7	65.98	7.52	3.17	78.76	7.35	13.89

**Table 2:** Textural parameters of NRPC-112 and CN800.

	SBET (m^2^ g^−1^)			Pore Volume (cm^3^ g^−1^)			
Material	Total	Micro	Meso	Total	Micro	Meso	Dp(nm)
CH-100	15	6	9	0.016	0.0018	0.0142	4.57
NRPC-112	2573	2398	175	0.89	0.78	0.11	1.99
C800	21	3	18	0.06	0.001	0.059	11.73
CN800	865	831	34	0.44	0.38	0.06	2.05

**Table 3:** Capacitances of NRPC-112 and CN800 in various current densities (F g^−1^).

Current Density (A g^−1^)	0.5	1	2	5	10
NRPC-112	340	329	316	287	273
CN800	246	216	189	158	111

**Table 4:** Adsorption capacity of NRPC-112 and CN800 in DCP, TCS and ROX.

Adsorbent	Adsorption Capacity (mg g^−1^)		
	DCP	TCS	ROX
NRPC-112	174	205	94
CN800	308	225	142
